# Rapid Manipulation in Irradiance Induces Oxidative Free-Radical Release in a Fast-Ice Algal Community (McMurdo Sound, Antarctica)

**DOI:** 10.3389/fpls.2020.588005

**Published:** 2020-11-25

**Authors:** Fraser Kennedy, Andrew Martin, Katerina Castrisios, Emiliano Cimoli, Andrew McMinn, Ken G. Ryan

**Affiliations:** ^1^ Institute for Marine and Antarctic Studies, University of Tasmania, Hobart, TAS, Australia; ^2^ Geography and Spatial Sciences, School of Technology, Hobart, TAS, Australia; ^3^ School of Biological Sciences, Victoria University of Wellington, Wellington, New Zealand

**Keywords:** oxidative stress, Antarctica, sea-ice algae, hydrogen peroxide (H_2_O_2_), nitric oxide (NO), microelectrodes, snow, photophysiology

## Abstract

Sea ice supports a unique assemblage of microorganisms that underpin Antarctic coastal food-webs, but reduced ice thickness coupled with increased snow cover will modify energy flow and could lead to photodamage in ice-associated microalgae. In this study, microsensors were used to examine the influence of rapid shifts in irradiance on extracellular oxidative free radicals produced by sea-ice algae. Bottom-ice algal communities were exposed to one of three levels of incident light for 10 days: low (0.5 μmol photons m^−2^ s^−1^, 30 cm snow cover), mid-range (5 μmol photons m^−2^ s^−1^, 10 cm snow), or high light (13 μmol photons m^−2^ s^−1^, no snow). After 10 days, the snow cover was reversed (either removed or added), resulting in a rapid change in irradiance at the ice-water interface. In treatments acclimated to low light, the subsequent exposure to high irradiance resulted in a ~400× increase in the production of hydrogen peroxide (H_2_O_2_) and a 10× increase in nitric oxide (NO) concentration after 24 h. The observed increase in oxidative free radicals also resulted in significant changes in photosynthetic electron flow, RNA-oxidative damage, and community structural dynamics. In contrast, there was no significant response in sea-ice algae acclimated to high light and then exposed to a significantly lower irradiance at either 24 or 72 h. Our results demonstrate that microsensors can be used to track real-time *in-situ* stress in sea-ice microbial communities. Extrapolating to ecologically relevant spatiotemporal scales remains a significant challenge, but this approach offers a fundamentally enhanced level of resolution for quantifying the microbial response to global change.

## Introduction

Light is the primary driver of ice-associated phototrophy in polar marine ecosystems and variation in the quantity and quality of *in-situ* irradiance significantly influences primary production (Arrigo et al., 2008; Petrou et al., 2016). When light penetrates sea-ice, it interacts with the crystal lattice structure causing significant refraction and exponential attenuation of photosynthetically active radiation (PAR: 400–700 nm) with depth. Due to its higher albedo and light scattering properties, snow often plays a significant role in limiting the irradiance reaching ice-associated microbial communities (Mundy et al., 2007). In land-fast ice, the majority of photosynthetic organisms are concentrated within 10–15 mm of the ice-water interface (Ryan et al., 2009; McMinn et al., 2010a) and bottom-ice microalgae are among the most shade-adapted organisms known to exist (Lizotte and Sullivan, 1991). Active growth has been recorded at <5 μmol photons m^−2^ s^−1^, while photoinhibition/saturation can occur at <20 μmol photons m^−2^ s^−1^ (Palmisano et al., 1987; McMinn et al., 1999, 2000). However, the mechanisms underlying efficient shade-adaptation can also limit the capacity for photosynthetic adjustment when sudden changes in irradiance occur (Raven, 2011; Raven and Beardall, 2011). The extreme variation of PAR in the sea-ice environment has forced many diatom species to evolve mechanisms that enable rapid adjustment of their photosynthetic apparatus to either increase photon capture in low light or minimize damage from excess irradiance (Mock et al., 2017; Kennedy et al., 2019). However, high inter-annual variability in irradiance (Turner et al., 2008) and the increased occurrence of extreme climatic events (Massom and Stammerjohn, 2010) in this region will influence both the timing and magnitude of light transmission and subsequently the development of ice-associated microbial communities (Deppeler and Davidson, 2017). The predicted loss of annual Antarctic sea ice (est. between 17–31%) within the next century will also influence the ecological integrity of the sea ice ecosystem. Advances in the application of microsensors that measure fluctuations in both the rate of and products relating to photosynthesis [e.g., oxygen (O_2_), glucose, hydrogen peroxide (H_2_O_2_), nitric oxide (NO)], could provide a real-time indication of cell health for effectively mapping the *in-situ* physiological status of ice-associated microbial communities.

Current climate models predict that the Antarctic ecosystem will undergo extensive cumulative environmental change as a result of continued anthropogenic-driven forcing (Bracegirdle et al., 2008; Turner et al., 2008). Projections indicate that less ice will form in warmer water during winter and melt earlier in spring/summer (Massom and Stammerjohn, 2010). These alterations in sea-ice environmental dynamics are likely to impact phototrophic organisms associated with the sea ice-matrix. Whether these changes will positively or negatively impact ice-associated primary production is currently unknown. However, microalgae are fundamental drivers of the Antarctic marine food-web (Ryan et al., 2006; Deppeler and Davidson, 2017) and any significant disruption in primary production is likely to impact trophodynamics. Furthermore, climate models infer an enhanced poleward shift in storm events resulting in higher winds, wave magnitude, and precipitation (Bracegirdle et al., 2008; Massom and Stammerjohn, 2010). Increased wind and wave action will intensify the fragmentation of existing sea-ice floes, accelerating ice melt leading to alterations in productivity. Increased precipitation as rain will enhance light transmission, whereas precipitation as snow will both decrease the quantity and alter the spectral composition of the irradiance reaching bottom-ice communities (Massom and Stammerjohn, 2010; Meiners et al., 2017). The overall predicted increase in PAR could lead to photodamage and oxidative stress in sea-ice algae, a dynamic which, in the Arctic, has been shown to stall *in-situ* primary production over timescales ranging from days (e.g., Juhl and Krembs, 2010; Lund-Hansen et al., 2014) to months (e.g., Campbell et al., 2015).

Environmental perturbation can influence multiple aspects of phototrophic metabolism and cause significant stress that manifests first in the photosynthetic apparatus (e.g., Murata et al., 2007; Ryan et al., 2009; Castrisios et al., 2018). While photosynthesis primarily serves to capture and convert light into cellular energy, it can also function as an efficient monitoring system for environmental change. During periods of significant environmental stress, alteration in the photosynthetic redox status and balance of photon capture to assimilation all act as molecular messengers that activate metabolic and photosynthetic adjustment. The timing and magnitude of change in irradiance is critical in determining the capacity for algal acclimation (Juhl and Krembs, 2010). On the scale of hours to days, bottom-ice algae can efficiently photoacclimate to increased irradiance by modifying their light-harvesting pigments and reaction centers (Katayama et al., 2011; Kuczynska et al., 2015). Acclimation on the scale of minutes to hours is also achievable but is constrained by a cell’s real-time photophysiological state (Juhl and Krembs, 2010; Petrou et al., 2010). The failure to quickly acclimate can rapidly lead to photoinhibition if available photo-protection mechanisms cannot efficiently dissipate excess excitation energy (Ruban et al., 2004). Photoinhibition alone is not necessarily damaging under normal circumstances and can even function as a repair mechanism by triggering the photo-inactivation of light-harvesting proteins (Murata et al., 2007; Ryan et al., 2009). However, under conditions of saturating irradiance, the rate of inactivation can exceed the rate of repair and prolong photoinhibition causing photodamage (Nishiyama et al., 2006). In these circumstances, the absorbed energy exceeds the capacity for utilization and electrons leak onto O_2_, generating harmful reactive oxygen species (ROS; Mallick and Mohn, 2000; Juhl and Krembs, 2010; Diaz et al., 2018; Huang et al., 2018). This leads to the formation of superoxide (O_2_^−^) free-radicals that can be converted into H_2_O_2_ and hydroxyl radicals (OH^−^). Under normal photosynthetic conditions, the level of these radicals is reduced to sustainable levels by efficient antioxidation mechanisms that limit damage. However, when exposed to excessive irradiance, ROS detoxification mechanisms can be insufficient, leading to ROS-induced cellular damage and the release of oxidative free radicals. Recently, Kennedy et al. (2020) showed that nitric oxide (NO) can be released in sea-ice algae during periods of environmental stress and that NO can significantly impact cellular growth and impair aspects of photosynthetic electron transport.

A non-destructive method that aims to measure the *in-situ* physiological status of under-ice algae communities is needed particularly during events that trigger environmental flux. In this study, microsensors were used to examine the influence of rapid shifts in irradiance on extracellular oxidative free radicals produced by sea-ice algae. Specifically, we aim to determine if (a) the photosynthetically coupled oxidative metabolites H_2_O_2_ and NO are produced following controlled manipulation of PAR at the ice-water interface and (b) whether these changes influence ice-algal biomass, community structure, photophysiology, and intracellular integrity.

## Materials and Methods

### Study Site and Experimental Design

Sea-ice algae concentrated in the bottom 300 mm of 1.8 m thick fast-ice at Cape Evans, McMurdo Sound, Antarctica (77°38S, 166°24E) were collected between November 11 and December 7, 2018. An area measuring ~20 × 20 m was selected in which nine 5 × 5 m plots were established (Supplementary Figure S1). These dimensions ensured that the under-ice microbial community at the center of each plot would be unaffected by light from outside the plot. The nine plots were randomly assigned to one of three treatments by placing snow to a specific depth (*n* = 3; Table 1). The under-ice microbial community was acclimated to the respective level of irradiance for 10 days prior to snow being either added or removed (referred to as “transitioned”), to rapidly alter the under-ice irradiance (Table 1). By manipulating snow depth, irradiance increased from 0.5 μmol photons m^−2^ s^−1^ in low light treatments (LL – 30 cm snow cover) to 13 μmol photons m^−2^ s^−1^ (HL – no snow) when snow was removed. Similarly, in high light treatments (HL – no snow), irradiance decreased from 13 to 0.5 μmol photons m^−2^ s^−1^ (LL – 30 cm snow) when snow was added. Control treatments (mid-range light – 5 μmol photons m^−2^ s^−1^ – 10 cm of snow) were not “transitioned”; snow cover was kept constant at 10 cm depth for the duration of the experiment. Plots allocated a HL (no snow) treatment were cleared of any settled snow throughout the day, to ensure maximum light penetration. Samples were extracted immediately before the transition and 24 and 72 h following the rapid alteration in irradiance.

**Table 1:** Under-ice irradiance was manipulated in plots measuring 5 × 5 m by placing snow to a specific depth: (1) low light – 0.5 μmol photons m^−2^ s^−1^ (30 cm snow), (2) mid-range light – 5 μmol photons m^−2^ s^−1^ [10 cm snow (control)], and (3) high light – 13 μmol photons m^−2^ s^−1^ (no snow cover).

Treatment before transition	Acclimation 10 days	Time after snow transition	
		24 h	72 h
Low light (snow – 30 cm)	→	High light	No Snow
Mid-light (control – 10 cm)	→	Mid-light	Mid-light
High light (no snow)	→	Low light	Low light

The under-ice microbial community was acclimated to the irradiance level for 10 days before snow being either added or removed (transition) to rapidly alter under-ice irradiance. Control treatments (mid-range light – 10 cm snow thickness) were not “transitioned”; snow cover was kept constant at 10 cm depth for the duration of the experiment. Samples for analysis extracted at 24- and 72-h following snow transition.

**Figure 1:**
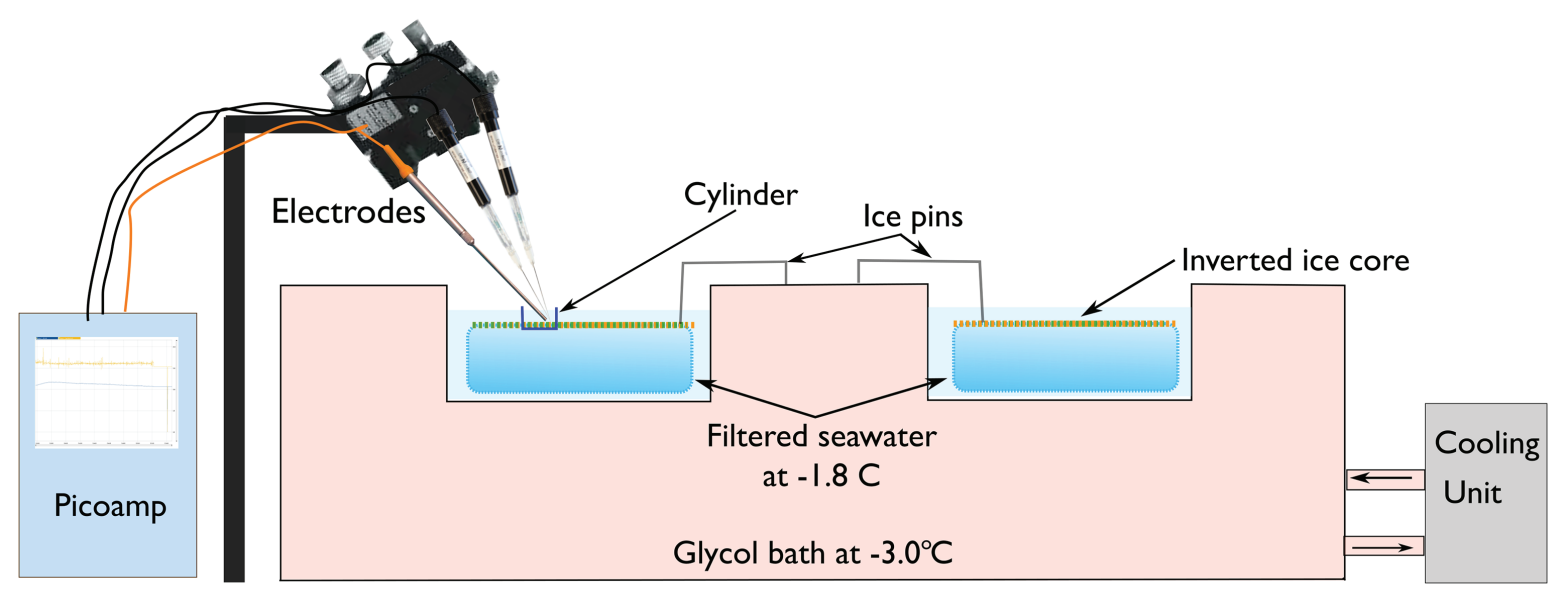
Ice cores were inverted and placed within individual chambers and then submerged in filtered seawater at a temperature of −1.8 ± 0.5°C. Three microelectrodes [hydrogen peroxide (H_2_O_2_), nitric oxide (NO), and oxygen (O_2_)] were manipulation in place above the inverted ice core containing the algal mat.

Ice cores were extracted from the center of each quadrat, three cores were used for microsensor experimentation, and one for species identification and intracellular assays. Cores were obtained by drilling to within 300–500 mm of the ice-water interface with a powered Jiffy ice auger, and cores 300–500 mm long were then extracted using a Mark V SIPRE ice corer (Kovaks, United States). The bottom section of the core was collected for analysis, the remaining section of the core (1,300–1,500 mm) and ice shavings were returned to the hole of extraction to limit light transmission. To minimize light shock, all cores were extracted under a black cover, wrapped in black plastic, and immediately transferred to a field laboratory (Ryan and Beaglehole, 1994). For I-PAM and microsensor analysis, the bottom 30 mm of each extracted core was cut into a 50 × 50 × 30 mm block, placed into a clear plastic container, and immersed in pre-chilled filtered seawater (−1.8°C); containers were partially submerged in a re-circulating ethylene glycol-filled water-bath maintained at −3.0 ± 0.5°C (Figure 1). Under-ice irradiance was measured at midday using a RAMSES radiometer (TriOS optical systems, Germany), which was attached to an articulating arm (McMinn and Ashworth, 2007). The radiometer was inserted through an adjacent ice hole and maneuvered to the center of each treatment (Supplementary Figure S1).

### Photophysiology, Chlorophyll *a*, and Species Composition

Chlorophyll *a* fluorescence was measured using a Maxi imaging pulse amplitude modulated fluorometer (I-PAM, Walz Germany) using the methods described by Castrisios et al. (2018). Briefly, extracted ice-cores were cut into 50 × 50 × 30 mm blocks, placed in black plastic containers, and carefully aligned into position on the I-PAM stage. The saturation pulse method was then used to determine the maximum quantum yield by measuring the minimum (Fo) and maximum (Fm) fluorescence of the inverted algae biofilm in a dark-adapted state (Castrisios et al., 2018). Fv/Fm is a sensitive indicator of photosystem potential and a proxy for cellular health in photosynthetic organisms. The area-of-interest (AOI) function was used to determine Fv/Fm of discreet locations on the ice core. Three AOIs were selected in areas where the minimum fluorescence (Fo) was >0.15. The process of fluorescence imagery was conducted within 30 s. Following imaging, samples were placed in a water-bath containing vessels of pre-chilled (−1.8°C) filtered seawater and left to equilibrate for 3 min in the dark before microsensor analysis.

Chlorophyll *a* (chl*a*) concentration was determined by collecting microalgal cells from within a cylinder embedded on the ice-surface of inverted ice-cores. Samples were filtered onto 25 mm GF/F filters and extracted in 10 ml of methanol in the dark at 4°C for 12 h. Chl*a* extracts were subsequently measured on a Turner fluorometer (10AU Turner designs, United States). Chl*a* concentration was determined in triplicate using the acidification protocol as described by Evans et al. (1987).

For species composition, a 2 ml sample was taken randomly from the bottom of an ice core collected from the center of each treatment and fixed in 0.5% glutaraldehyde. A 0.5 ml aliquot from each sample was prepared using a settling chamber and cells were examined using a Nikon eclipse Ci (Nikon Corp, Australia). Whole-cell microalgae were counted from 10 randomly selected fields of view and taxonomically identified using the descriptions in Scott and Marchant (2005). A minimum of 100 cells were counted per sample.

### The Measurement of Extracellular Oxidative Free Radicals

The pre-cut ice cores were inverted in the field laboratory so that the algal mat faced upwards (Figure 1). The temperature of the ice-core was maintained at −1.8 ± 0.5°C by floating the plastic containers in a refrigerated recirculating water-bath containing ethylene glycol at −3.0 ± 0.5°C. To ensure ice cores did not float and damage the microsensors, cores were kept submerged by two spring-loaded metal pins (Figure 1). A clear plastic cylinder measuring 1 × 1 cm was placed in the center of each ice-core to ensure that the measurement of extracellular metabolites was isolated to discreet patches (Figure 1). The under-ice irradiance of each treatment was simulated in the laboratory using a Lumitronix P1-500-VIS-IR growth lamp (Futureled GmbH, Germany). This lamp provides full PAR coverage (>400–700 nm) plus 730 nm. It must be noted that while this light source is close to natural sunlight, which is not a perfect representation; there is a slight peak in the UV-B range and UV-A is lacking. To ensure that illumination was consistent across the samples, irradiance was adjusted using neutral density filters to correspond with the *in-situ* under-ice irradiance (Table 2).

**Table 2:** The mean relative species composition (10^4^ cells L^−1^) of bottom-ice alga subjected to three irradiances (1) low light (0.5 μmol photons m^−2^ s^−1^), (2) mid-range (5 μmol photons m^−2^ s^−1^), and (3) high light (13 μmol photons m^−2^ s^−1^).

Species	Under-ice irradiance								
	Before transition			24 h			72 h		
	Control	Low	High	Control	Low → High	High → Low	Control	Low → High	High → Low
*Nitzschia stellata*	28.2 [2.5]	**21.4** [2.3]	38.5 [2.7]	**31.5** [6.6]	**29.1** [8.2]	**18.4** [3.9]	**11.5** [2.5]	21.7 [5.6]	**17.3** [1.9]
*Berkeleya adeliensis*	24.2 [1.5]	11.2 [1.7]	**60.4** [6.8]	17.3 [4.0]	11.1 [4.8]	16.6 [2.9]	7.2 [1.1]	20.7 [4.9]	4.0 [0.8]
*Entomoneis kjellmannii*	2.8 [0.3]	4.8 [1.9]	6.6 [0.07]	5 [4.0]	0.9 [0.1]	0.7 [0.1]	0.3 [0.3]	6.9 [2.0]	0.5 [0.4]
*Fragilariopsis* sp.	4.8 [0.1]	6.6 [0.2]	6.6 [0.3]	1.7 [1.1]	2.3 [0.4]	0.9 [0.4]	1.5 [1.3]	5.7 [0.6]	2.1 [0.4]
*Nitzschia sp.*	4 [0.3]	4.4 [0.1]	6.4 [0.1]	0.9 [0.3]	0.9 [0.4]	0.4 [0.1]	1.7 [0.9]	3.5 [1.1]	1.5 [0.7]

Before transition represents the respective treatment allocated before the shift in light level. Following a 10-day acclimation period, the snow cover was “transitioned” as previously described (Table 1). Numbers in bold highlight apparent dominant species of the community. Values in square brackets describe the SE (10^4^) associated with each species count.

Ice cores were extracted from the quadrats sequentially to minimize the delay (maximum delay in processing 2–3 min) in collecting microsensor data. Cut cores were left to equilibrate in the water bath in the dark for 3 min. Samples were then exposed to their respective light regime for 6 min during which time, H_2_O_2_, NO, and O_2_ concentration was measured. Microsensors were aligned to within 100 μm of each other and inserted to within 50 μm of the algal biofilm using a motorized micro-manipulator. Each microsensor was calibrated before use in filtered (0.45 μm), pre-chilled (−1.8°C) seawater as per the manufacturer’s instructions. The sensors were left to equilibrate for 1 min before logging the electrochemical trace over a total of 6 min. H_2_O_2_ (μmol), NO (nmol), and O_2_ (μmol) concentrations were determined by extracting the last 5 min of each electrochemical trace and calculating the mean; the results were then normalized to the chl*a* concentration of each sample. Chl*a* is the primary photosynthetic pigment utilized by Antarctic sea-ice algae and is the accepted proxy for estimating algal biomass (Meiners et al., 2012, 2018). Because the amount of chl*a* is proportional to cell size, data were normalized to chl*a* rather than cell counts.

Hydrogen peroxide concentration was measured using electrodes obtained from World Precision Instruments (HPO, tip diameter – 2 mm WPI, United States), these were connected to a WPI free radical analyzer. O_2_ and NO were measured with Clarke-type electrodes (O_2_, tip diameter – 50 μm; NO, tip diameter – 100 μm; Unisense, A/S, Aarhus, Denmark) connected to a field multimeter (Unisense, A/S, Aarhus, Denmark).

### Oxidative RNA Damage

To determine if oxidative free radicals can cause damage to intracellular molecules, an oxidation RNA damage ELISA kit was used (Cell Biolabs, Inc. Sand Diego, CA, United States). This assay detects the formation of 8-hydroxyguanosine (8-OHG), which is a biomarker for oxidative damage. Calculation of 8-OHG concentration was conducted fluorometrically on a microplate reader (BMG FLUOstar OPTIMA, Mornington, VIC, Australia) using the manufacturer’s instructions. 8-OHG levels were normalized to the total RNA concentration of each sample. RNA was initially extracted using a RNeasy Mini Kit (Qiagen, Australia) supplemented with molecular grinding resin (Gene Technology, United States) to maximize RNA yield.

### Statistical Analysis

Where appropriate, data were analyzed using two-way repeated measures Analysis of Variance (ANOVA) to examine variation among treatments (snow loading) over time using the software package R [R core team (2020), version 1.3.1093]. Data were checked for normality by the Shapiro-Wilk test and sphericity using Mauchly’s test using the R function anova_test(). If significant differences were detected, *post-hoc* pairwise *t*-tests were conducted on Bonferroni adjusted values of *p* (*p* < 0.05) to determine the influence of the main effect (snow loading) had on oxidative free-radical release over time (*n* = 3). Data are expressed as mean ± 1 SD unless otherwise stated.

Pearson correlations were performed to observe the relationship between different extracellular oxidative species. The final 60 s of each electrochemical trace (*n* = 3) was used for this analysis. Means of the traces were used to determine the significance of correlations.

## Results

**Figure 2:**
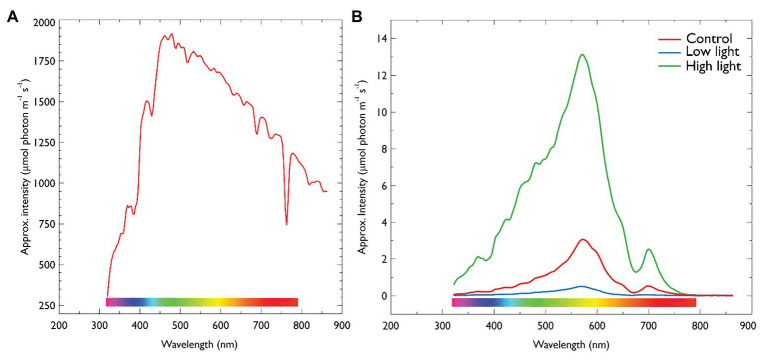
**(A)** Irradiance on ice-surface collected at midday. **(B)** Midday under-ice irradiance of each treatment described in Table 1. Irradiance was collected using a RAMSES radiometer (TriOS optical systems, Germany). Values are approximate intensities in μmol photons m^−2^ s^−1^. For under-ice measurements, the radiometer was attached to an articulating arm and inserting through an adjacent ice hole and maneuvered into center of each treatment. Control = 5 μmol photons m^−2^ s^−1^, 10 cm snow; Low light = 0.5 μmol photons m^−2^ s^−1^, 30 cm snow cover; High light = 13 μmol photons m^−2^ s^−1^, no snow.

The annual fast-ice at Cape Evans in 2018 was 1.8 m thick and contained a community dominated by diatoms at the ice-water interface (Table 2). Snow-cover effectively altered the intensity of PAR reaching the ice-water interface (Figures 2A,B).

### Electrochemical Measurement of Extracellular Oxidative Free Radicals

**Figure 3:**
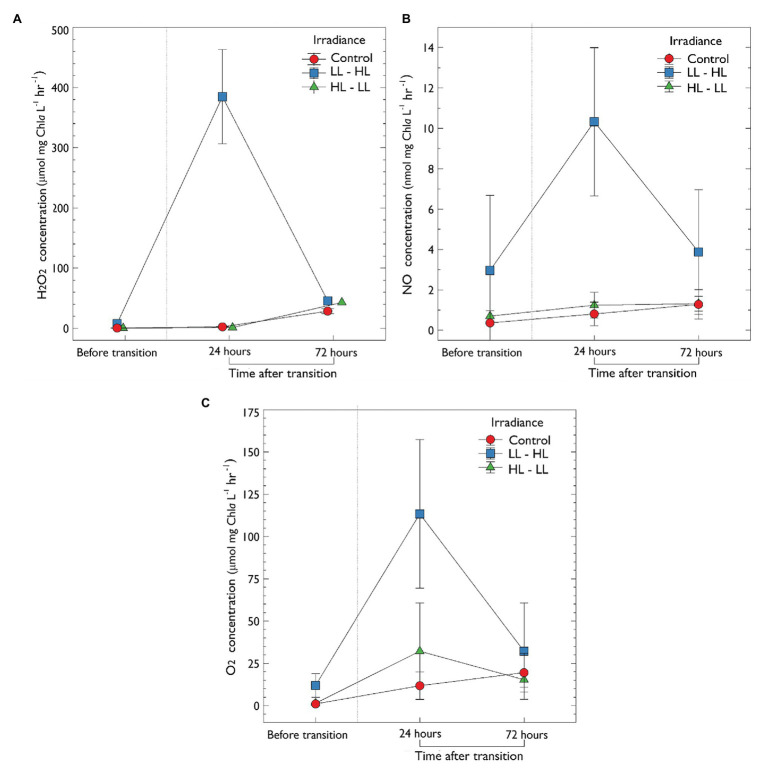
*In-vitro* production of extracellular metabolites determined by microsensors of ice-water interface algal exposed to one of three irradiance levels (1) LL – low light [0.5 thick snow (30 cm)], (2) mid-range – control, and (3) HL – high light. **(A)** Concentration of extracellular H_2_O_2_ (μmol mg chl*a* L^−1^). **(B)** Nitric oxide concentration (nmol mg chl*a* L^−1^). **(C)** Oxygen concentration (μmol mg chl*a* L^−1^). Sea-ice algal communities were acclimated to their respective snow treatment for 10 days before sampling. After 10 days, an initial sample was collected, denoted “before transition”; following the acclimation period, irradiance either increased or decreased (*via* manipulation of snow-depth) as previously described. Samples were then collected at 24 and 72 h after the transition. Control, no change in irradiance; LL – HL, low light to high light; HL – LL, high light to low light. Error is the standard deviation of the mean (*n* = 3).

Hydrogen peroxide production was successfully measured for the first time in sea-ice cores. Following the removal of snow from low light plots (LL → HL), a significant increase in H_2_O_2_ concentration was observed from 0.71 ± 0.45 before snow removal to 362.89 μmol H_2_O_2_ mg chl*a* L^−1^ h^−1^ 24 h after (*F*_2, 18_ = 30.424, *p* < 0.001, *post-hoc* pairwise *t*-test Bonferroni adjusted, *p* < 0.001). After 72 h, H_2_O_2_ production in LL → HL plots reduced to a mean of 13.23 μmol H_2_O_2_ mg chl*a* L^−1^ h^−1^, which was not significantly different compared to the other treatments (Figure 3A). There was also no significant difference in H_2_O_2_ production in treatments transitioning from high light (no snow) to low light (30 cm snow; HL → LL) compared to the control (mid-range – 10 cm snow). In the control treatments, no significant increase in H_2_O_2_ was observed during the experiment.

The oxidative metabolite NO was also measured electrochemically for the first time in a sea-ice community. Following the removal of snow from low light plots (LL → HL), there was a significant increase in the concentration of NO from 2.97 ± 1.22 SD to 10.32 ± 3.66 nmol mg chl*a* L^−1^ h^−1^ 24 h after the increase in irradiance (*F*_2,18_ = 3.571, *p* < 0.001, *post-hoc* pairwise *t*-test Bonferroni adjusted, *p* < 0.05; Figure 3B). Following 72 h, NO concentration decreased to non-significant levels (3.874 ± 3.04 nmol mg chl*a* L^−1^ h^−1^. There was no statistically significant difference in NO concentration during the transition from high to low light (HL → LL) when compared to the control (*p* > 0.05; Figure 3B).

A statistically significant increase in O_2_ concentration was observed in treatments that transitioned from low to high light (LL → HL; from 12.14 ± 4.87 SD to 113.39 ± 43.94 μmol mg chl*a* L^−1^ h^−1^; *F*_2,18_ = 32.13, *p* < 0.05, *post-hoc* pairwise *t*-test Bonferroni adjusted, *p* < 0.001; Figure 3C). Following 72 h at this new light treatment, O_2_ concentration decreased in this treatment (to 32.18 ± 28.54 μmol mg chl*a* L^−1^ h^−1^) although this change was not significant (*p* > 0.05). There was also no significant difference in O_2_ concentration when comparing the control (mid-range light) and high to low light treatment (*p* > 0.05; Figure 3C).

To determine if the increase in H_2_O_2_ observed in low to high light was related to O_2_ production, values were correlated using the Pearson correlation coefficient (Supplementary Figure S2). A statistically significant difference was observed 24 h after the increase in irradiance from low to high light (Pearson correlation coefficient of 0.79; *R*^2^ = 0.62, *p* < 0.001 (Supplementary Figure S2). There was no relationship between H_2_O_2_ and O_2_ before the changes in irradiance (*R*^2^ = 0.002, *p* = 0.81), and 72 h after (*R*^2^ = 0.012, *p* = 0.58; Supplementary Figure S2). There was no significant correlation between NO and the production of both H_2_O_2_ and O_2_. Additionally, no significant correlation in the production of metabolites was observed in the control (mid-range) and high to low light treatments.

#### Species Composition

In treatments before snow-cover manipulation, the colonial pennate diatom *Nitzschia stellata* dominated communities exposed to low irradiances and was marginally higher in the control. In treatments exposed to HL (no snow) for 10-days, the tube-dwelling diatom, *Berkeleya adeliensis*, was more abundant (Table 2). In the 24 h following changes in under-ice irradiance, *N. stellata* was dominant in all treatments irrespective of the light level. After 72 h, both *B. adeliensis* and *N. stellata* were highly represented under HL, and *N. stellata* appeared to be more prominent in communities exposed to low and mid-range irradiances (Table 2).

#### Chlorophyll *a*

**Figure 4:**
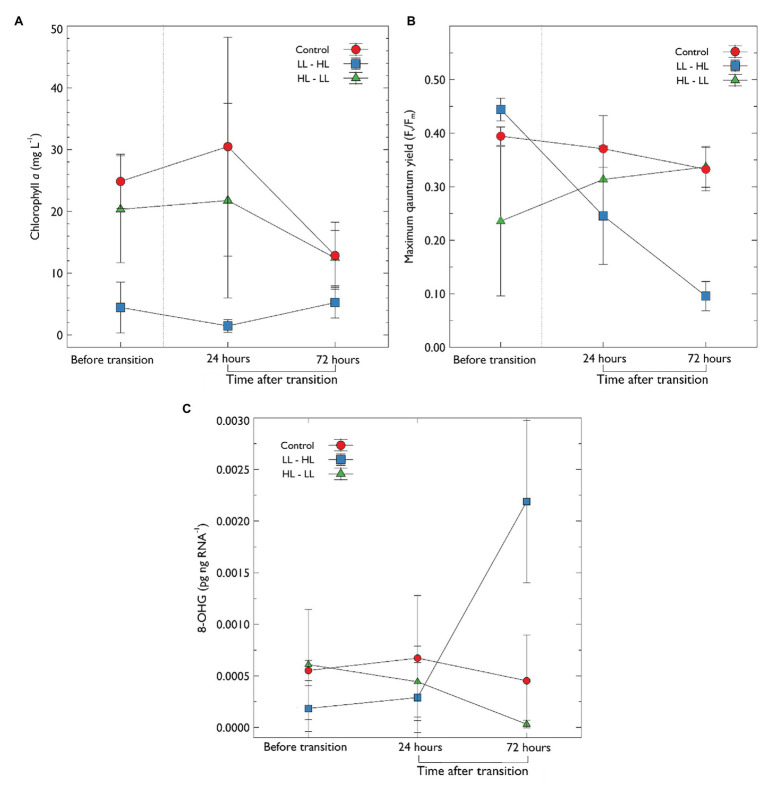
**(A)** Chlorophyll *a* concentration and **(B)** maximum quantum yield (Fv/Fm) of photosystem II and, **(C)** concentration of 8-OHG in algal extracts, 8-OHG is a marker for RNA oxidative damage. Sea-ice algal communities were acclimated to their respective snow treatment for 10 days before sampling. After 10 days, an initial sample was collected, denoted “before transition”; following this, the snow was transitioned as previously described (Table 1). Samples were then collected at 24 and 72 h after the transition. Error bars represent the standard deviation of the mean (*n* = 3).

After the 10 day acclimation period, chlorophyll *a* (chl*a*) concentration was significantly lower in treatments with low light (4.44 ± 4.10 mg chl*a* L^−1^) compared to both the control (24.86 ± 4.27 mg chl*a* L^−1^) and high light treatments (20.32 ± 8.64 mg chl*a* L^−1^; Figure 4A). In treatments following removal of thick snow chl*a* concentration did not significantly increase (72 h; 5.22 ± 2.50 mg chl*a* L^−1^). In comparison, in treatments transitioned from high to low light (HL → LL) chl*a* decreased by 38% after 72 h, although this was not significant (Figure 4A). Further analysis was performed to examine if chl*a* was correlated with snow depth (Supplementary Figure S3); the results indicate a negative correlation (Pearson correlation coefficient −0.7, *p* = 0.035, *R* ^2^ = 0.49) between chl*a* and snow depth in treatments before the change in irradiance transition. There was no significant correlation between snow depth and chl*a* following the transition.

The rapid transition from LL → HL, resulted in a statistically significant reduction in maximum quantum yield of PSII (Fv/Fm) from a mean of 0.444 ± 0.02 to 0.095 ± 0.027 after 72 h (*F*_2, 18_ = 4.856, *p* < 0.001, pairwise *t*-test Bonferroni adjusted *p* < 0.0001; Figure 4B). While Fv/Fm declined, the concentration of chl*a* did not change. In contrast, Fv/Fm improved in the high light to low light treatment (HL → LL); Fv/Fm increased from a mean of 0.235 ± 0.139 to 0.337 ± 0.03, however, this was not statistically significant (*p* > 0.05). Fv/Fm in the control treatment declined slightly (Figure 4B) but this was not statistically significant (*p* > 0.05).

#### Photophysiology

**Figure 5:**
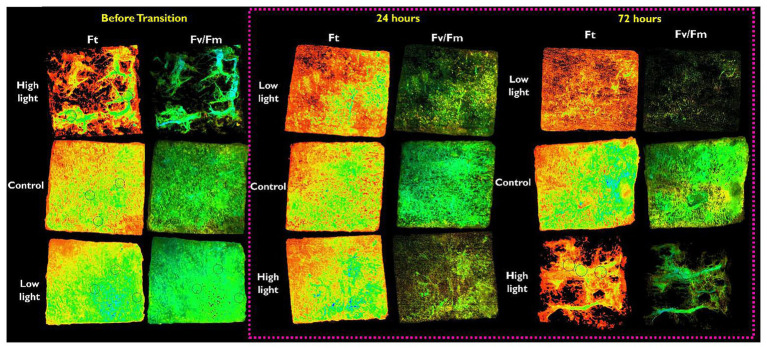
Representation of the fluorescent parameters obtained from an imaging-PAM (Walz, Germany). The figure shows a representative ice core cut to 50 × 50 mm extracted from the ice-water interface of each light treatment; low, mid-range (control), or high light. The ice cores are shown inverted so that the algal-mat from the ice-water interface is facing up. Images are grouped by the time after the transition (before transition, 24 h, or 72 h). The parameter Ft shows the distribution of the algal mat, the color green/blue represents areas of increased biomass. Black circles (visible in some images) within the images are area-of-interest (AOI) functions used to determine Fv/Fm (Figure 4B).

The I-PAM can provide distinct 2D images of the distribution of chl*a* fluorescence and physiological activity of photosystem II. A clear difference in the distribution of algal cells was observed after 72 h (Figure 5). Under-ice algal communities exposed to LL before the transition displayed a distinctly patchy distribution (as indicated by the variation in Ft) and a significant reduction in photosynthetic performance (Fv/Fm) was observed (Figure 4B). Interestingly, 24 h following increased irradiance in LL→ HL treatments, the patchy distribution changed to reflect a homogeneous nature over the ice-water interface. In contrast, when irradiance was reduced in HL → LL treatments, the relatively homogeneous algal mat evolved to form a patchy distribution (Figure 5). It must be noted that the I-PAM measures the total community response; it is not possible to distinguish between species using this technique. In addition, PAM provides a qualitative assessment of photosynthetic health; these data track a fluorescence signature that reflects community-level changes in species composition and relative changes in photosynthetic health.

#### Oxidative Damage of RNA

There was a statistically significant increase in the marker for RNA oxidative damage (increase in OHG) in LL → HL treatment at 72 h when compared to the mid-range light (LL → HL; *F*_(2, 18)_ = 7.273, *p* < 0.05) and HL → LL treatments (*p* < 0.002). There was no significant difference in the marker for RNA oxidative damage in any other treatments over time (Figure 4C).

## Discussion

Rapid oscillations in light level can trigger large variations in the degree of under-algal biomass in polar habitats (Palmisano et al., 1985; Ackley and Sullivan, 1994; Campbell et al., 2015; Leu et al., 2015; Wongpan et al., 2018). The timing and magnitude of PAR is critical in determining the extent of change and the severity of *in-situ* physiological stress (Mundy et al., 2005;Juhl and Krembs, 2010; Vermuelen, 2013). Rapid changes from low to higher irradiance may cause photoinhibition and impair phototropism in shade-adapted taxa (Campbell et al., 2015; Leu et al., 2015). In contrast, exposure to moderate-range PAR with limited change can improve photosynthetic performance (Lund-Hansen et al., 2014) and increase algal standing stocks (Wongpan et al., 2018). Importantly, the net response of bottom-ice algae to rapid changes in irradiance may be dependent on the ability to photoadapt coupled with the degree of change experienced (Juhl and Krembs, 2010). The results presented in this study confirm this relationship, whereby an inverse correlation between snow depth and chl*a* was present when the algal mat had sufficient time for acclimation (10 days). Furthermore, the community composition differed with irradiance level; *B. adeliensis* appeared to dominate when exposed to high light, whereas *N. stellata* was more prominent in both low and mid-range irradiances. In treatments acclimated to low light, the subsequent exposure to high irradiance resulted in a ~400× increase in the production of H_2_O_2_ and a 10× increase in NO concentration after 24 h. This is the first study to observe a real-time response of H_2_O_2_ and NO as a function of irradiance in an Antarctic sea ice microbial community.

Following the removal of thick snow, there was a rapid decline in the photosynthetic parameter Fv/Fm suggesting that cells were undergoing photo-physiological stress as a result of the increase in irradiance. Most records of the light saturation of photosynthesis of bottom-ice associated algae are <10 μmol photons m^−2^ s^−1^ (Palmisano and Sullivan, 1983; McMinn et al., 2003; Ryan et al., 2009), with photoinhibition often occurring at levels below 20 μmol photons m^−2^ s^−1^. During this experiment, the under-ice irradiance did not exceed 14 μmol photons m^−2^ s^−1^, however, rapid exposure to this irradiance appeared to negatively influence the photosynthetic capacity of the low-light adapted community. Furthermore, the decline in Fv/Fm (24 h) was correlated with an increase in the production of H_2_O_2_ and NO, suggesting that significant photo-oxidative stress was evident which may have contributed to the decline of the dominant species. The uncharged nature of H_2_O_2_ ensures easy diffusion across cell membranes, which under saturating conditions can result in an extracellular overflow (Ivanov et al., 2018). High concentration of H_2_O_2_ has been reported within Antarctic sea-ice, particularly in the upper sections where exposure to high irradiance is common (Cooper and Zika, 1983; Neftel et al., 1984; King et al., 2005). The half-life of H_2_O_2_ in seawater is prolonged (Wong et al., 2003), and it can remain for up to 15 days in polar waters (King et al., 2005). This may have a considerable influence on under-ice biology as the toxicity of H_2_O_2_ is variable among taxa. Florence and Stauber (1986) showed that species of *Nitszchia* remain highly susceptible to oxidative damage in the presence of H_2_O_2_ in the immediate environment. In contrast, Drábková et al. (2007) showed that the diatom *Navicula* could tolerate relatively high levels of H_2_O_2_. This reduction in H_2_O_2_ may indicate that cells had photo-acclimated to the incident irradiance or illustrate that the species succession had shifted to a taxon favoring higher light (*B. adeliensis*). Furthermore, a similar pulse in NO was observed following exposure from low to high light (24 h). This correlated with the pulse in H_2_O_2_ and may also be explained in the breakdown of photosynthetic electron transport. The production of NO in the sea-ice diatom *Fragilariopsis cylindrus* occurs only in the dark or when the photosynthetic chain is inhibited (Kennedy et al., 2020). Under conditions of normal photosynthetic electron flow, the chloroplast enzyme nitrite reductase catalyzes the conversion of nitrite to ammonium. Essential to this reaction is the reduced form of ferredoxin, created when water is oxidized at photosystem II (PSII) to NADP+ during normal photosynthetic electron flow (Kennedy et al., 2020). So, when photosynthesis is operating normally, nitrite is rapidly assimilated and does not accumulate in the chloroplast. But when the supply of reduced ferredoxin diminishes, during conditions that disrupt normal photosynthetic electron flow (or in the dark), nitrite accumulates, causing the enzyme nitrate reductase to convert nitrite to NO (Dolch et al., 2017; Kennedy et al., 2020). The heightened production of NO observed here may further indicate that the shift in irradiance was sufficient to cause oxidative stress and inhibition of normal photosynthetic electron flow in shade-adapted algae. Extreme levels of ROS damage the photosynthetic apparatus, prolong photoinhibition, inhibit the Calvin-Benson cycle, cause DNA damage, and stall active growth (Nishiyama et al., 2006; Murata et al., 2007; Janknegt et al., 2008; Rijstenbil, 2010). It is surmised here that the rapid increase in irradiance caused significant production of ROS, whereby it affected the physiology of the algal community through photodamage and photoinhibition and temporarily compromised intracellular integrity with respect to RNA damage.

Diatoms dominate the ice-water interface of fast ice and are highly shade-adapted. Active growth has been recorded at very low radiances (<5 μmol photons m^−2^ s^−1^) in both the Antarctic (McMinn et al., 2010b) and the Arctic (Hancke et al., 2018). In this study, the presence of snow significantly reduced the incident level of PAR reaching the ice-water interface. These changes in PAR had a significant influence on the community structure of bottom-ice associated microalgae taxa. In HL treatments, the tube-forming *B. adeliensis* was highly abundant, whereas the colonial pennate diatom *N. stellata* was the dominant species under low and mid-range irradiance. This pattern of community change in response to variation in light has been noted previously in land-fast ice in McMurdo Sound (Grossi and Sullivan, 1985; McMinn, 1997; Ryan et al., 2006; Vermuelen, 2013). Ryan et al. (2006) illustrated the species succession of *B. adeliensis* in communities exposed to higher light. McMinn (1997) observed the dominance of *N. stellata* in lower irradiance. More recently, Vermuelen (2013) also showed a change in the ratio of *N. stellata* to *B. adeliensis* with snow depth and concluded that *B. adeliensis* preferred higher light than *N. stellata*. Before the change in irradiance, a higher level of biomass was present under control plots (thin layer of snow) and less under low light plots (thick snow). Conversely, the maximum quantum yield of PSII (Fv/Fm) was higher when exposed to low light and considerably less in plots with high irradiance. Moreover, alterations in the distribution of the algal mat at the ice-water interface (I-PAM) were consistent with irradiance-induced shifts in species composition. This suggests that the community (dominated by *B. adelienis*) exposed to HL was either photophysiologically stressed or that photosynthetic capacity was reduced as a mechanism to limit photodamage (Kirst and Wiencke, 1995). Upon reduction of irradiance (HL – LL), Fv/Fm increased and the concentration of chl*a* declined, although this was not significant. It must be noted that the use of chl*a* (Fo) as a measure of biomass is viewed with caution because ice-associated taxa can rapidly modulate their pigment composition in response to irradiance (Mock et al., 2017; Kennedy et al., 2019). Thus, the decrease in chl*a* may be partially explained by the ability of diatoms to reduce the ratio of chl*a* to carotenoid pigments in response to the narrowing of the light spectrum (Mundy et al., 2005; Massom and Stammerjohn, 2010; Campbell et al., 2015). Chlorophyll *a* has light absorption peaks at 430 and 662 nm and the application of thick snow ensured significant attenuation of these spectral bands. Under this scenario, ice-algae may increase the production of higher wavelength absorbing carotenoid pigments as a mechanism to enhance the probability of photon capture (McMinn et al., 1999; Lizotte, 2001; Lyon and Mock, 2014; Kennedy et al., 2020).

There is a clear knowledge gap in our understanding of how sea-ice microbial communities will adapt to increasing environmental stress. This reflects not only the predicted loss of annual sea ice but the increase in extreme weather events that will exacerbate the magnitude of snow cover removal (wind) and deposition (wind, snow) events during spring. As a result, seasonal variation in spectral intensity and wavelength will intensify. In principle, we show that deeper snow will reduce light stress and enhance microbial growth, and this could mitigate the seasonal photoinhibition that would otherwise occur because of thinner ice. However, our data also illustrate the negative short-term effects of exposure to relatively high light associated with the oscillation frequency of snow loading. Because our experimental design reflects the constraints of deep-field Antarctic research, extrapolation to large regional and temporal scales would be overly simplistic, however, we clearly demonstrate that advances in the application of microsensors warrants future research. A predictive understanding of how shifts in ecosystem drivers in the Antarctic will impact sea-ice primary production will require an experimental framework that couples *in-situ* sensing technology with studies at ecologically relevant spatiotemporal scales. Bridging the gap between micro- and macro-scale perspectives in sea-ice microbial ecology is considered non-trivial, but the development of bio-platforms that enable long-term monitoring represents an exciting challenge in that regard. Future studies would also benefit from characterizing the secondary photosynthetic pigments ulilized by sea-ice algae, and correlating extracellular measurements of ROS to the intracellular processes that stimulate their production.

In summary, our results demonstrate that microsensors offer an enhanced level of resolution for tracking real-time *in-situ* stress in sea-ice microbial communities. This aligns with a fundamentally important goal of assessing the current state of Antarctic ecosystems and generating a baseline against which future change in ecosystem structure and function can be measured.

## Data Availability Statement

The raw data supporting the conclusions of this article will be made available by the authors, without undue reservation.

## Author Contributions

FK collected and analyzed the samples and wrote the manuscript. AMa and KR wrote the grant, designed the research, collected the samples, and assisted with the manuscript. KR and KC collected the samples and assisted with the manuscript. EC collected the under-ice irradiance data. AMc assisted with the manuscript. All authors contributed to the article and approved the submitted version.

### Conflict of Interest

The authors declare that the research was conducted in the absence of any commercial or financial relationships that could be construed as a potential conflict of interest.
