# Supplementary material for: Rapid Manipulation in Irradiance Induces Oxidative Free-Radical Release in a Fast-Ice Algal Community (McMurdo Sound, Antarctica)
Source: Front Plant Sci. 2020 Nov 25;11:588005. doi: 10.3389/fpls.2020.588005 (PMC7723870; doi:10.3389/fpls.2020.588005)
Supplement: Supplementary file 1 [file Data_Sheet_1.pdf]

Supplementary Material

1 Supplementary Figures

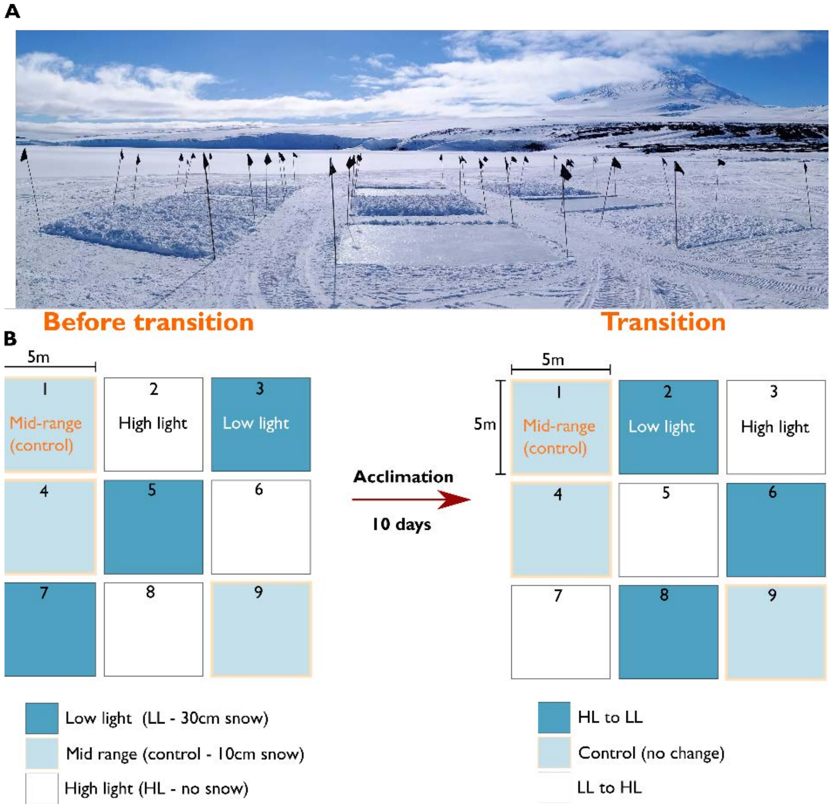

Figure S1: **A)** Photograph of the nine 5 m x 5 m treatments before the change in irradiance. **B)** schematic representation of the random allocation of snow treatments. Plots ("before transition") were acclimated to the respective irradiance level for 10 days before snow was removed or added ("transitioned") to alter the under-ice irradiance.

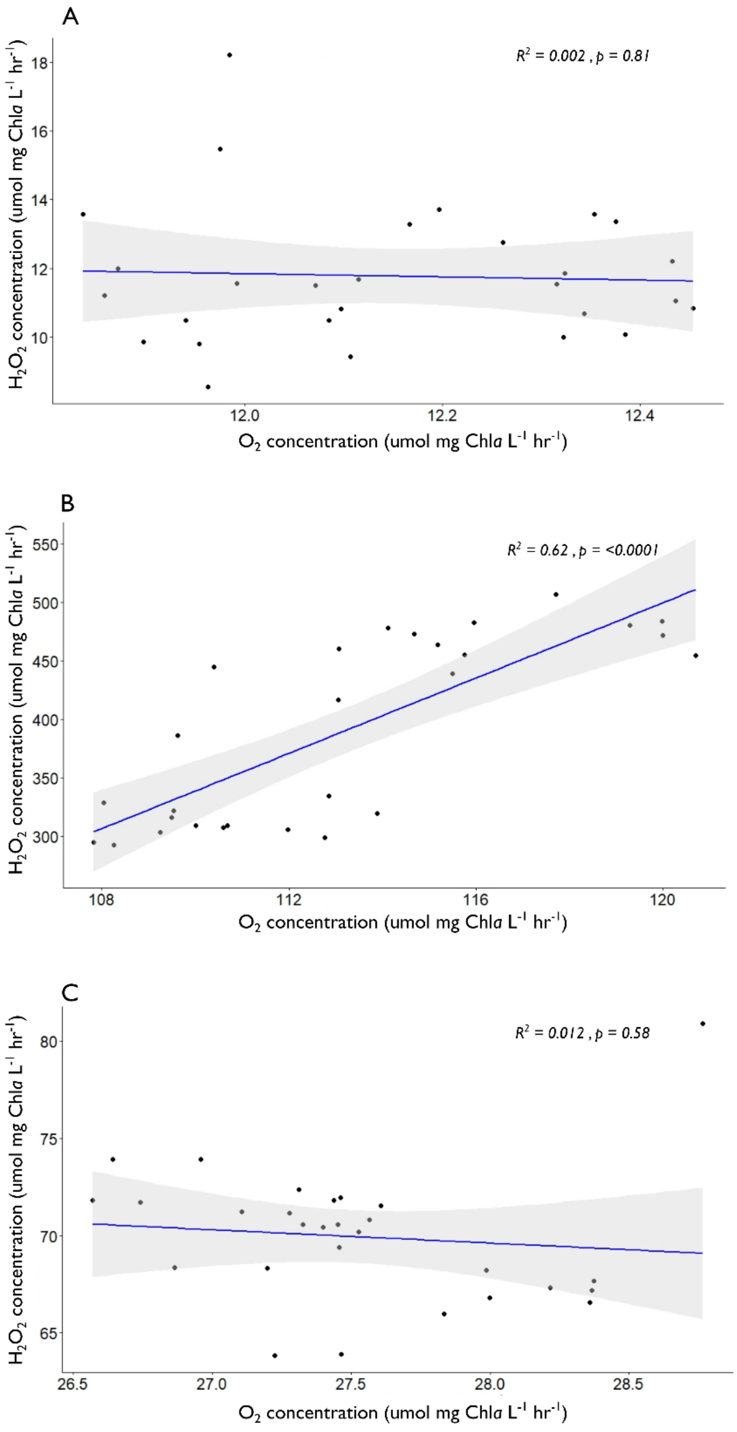

Figure S2: Pearson correlations in LL – HL treatments between H_2O_2 concentration (umol mg Chla L^{-1} hr^{-1}) and O_2 concentration (umol mg Chla L^{-1} hr^{-1}) over time. The final 60 seconds of each electrochemical trace (n = 3) was used for this analysis. Means of the traces were used to determine

the significance of correlations. **A)** before the transition (at LL treatment for 10 days before removal). **B)** 24 hours following the removal of snow loading and, **C)** 72 hours following transition.

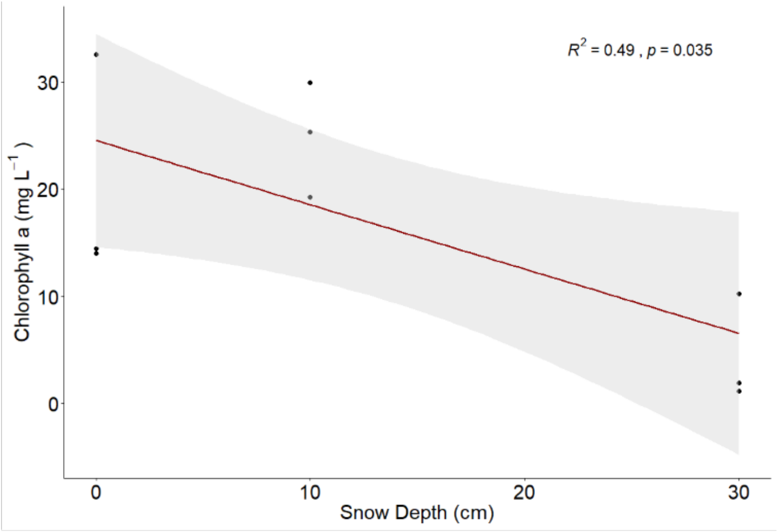

(a)

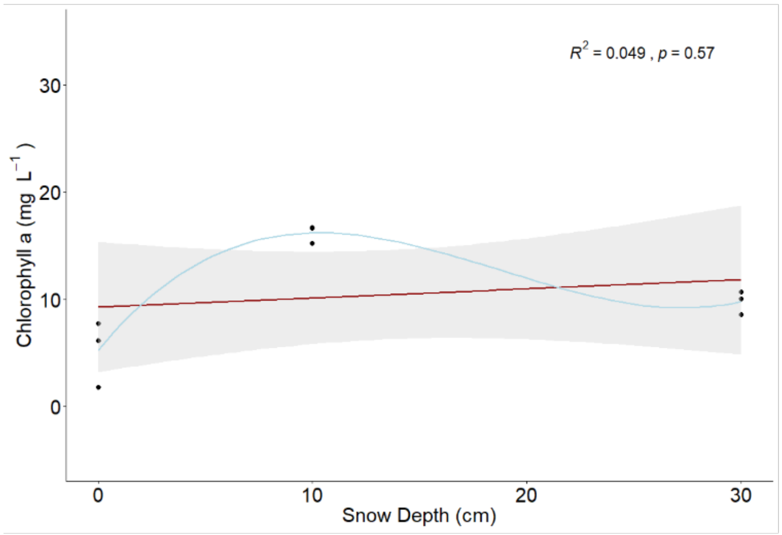

(b)

Figure S3. Correlation between chlorophyll a and snow thickness, **a)** before the transition (at snow thickness for 10 days before removal) and **b)** 72 hours after the reversal in the snow (either added or removed).
